# Half-Saline *versus* Combined Normal Saline and 1/3–2/3 Intravenous Fluid Therapy in Kidney Transplantation

**Published:** 2011-08-01

**Authors:** A. Ghorbani, M. Feli, A. Ehsanpour, H. Shahbazian, F. Hayati, J. Roozbeh

**Affiliations:** 1 *Departments of Nephrology, Golestan Hospital,*; 2 *Departments of Internal Medicine, Golestan Hospital, *; 3 *Department of Internal Medicine, Razi Hospital, *; 4 *Department of Nephrology, Imam Khomeini Hospital, Jundishapur University of Medical Sciences, Ahwaz, Iran,*; 5 *Shiraz Transplantation Research Center, Shiraz University of Medical Sciences, Shiraz, Iran*

**Keywords:** Renal transplantation, Intravenous fluid therapy, Renal function

## Abstract

Background: Sufficient intravascular volume should be established for optimal graft function after renal transplantation. However, there is no recommendation for the type of fluid therapy post-operatively. We compared half-saline *vs*. normal saline and 1/3–2/3 intravenous fluid replacement after renal transplantation.

Methods: We enrolled all patients who underwent kidney transplantation between June 2008 and March 2010 in Golestan Hospital, Ahwaz, southwestern Iran. Patients were randomly divided into two groups using a blinded allocation technique. Group A patients (Case) received half saline, and group B patients (Control) received normal saline and 1/3–2/3 intravenous fluid. According to our protocol, we replaced as much as 100% of hourly urine output in the first day, followed by 90% and 70% of every 2-hour urine output in the 2nd and 3rd days, respectively. Blood pressure and pulse rate were recorded hourly. Serum sodium, potassium, creatinine and pH were assessed twice a day.

Results: There were 34 and 36 eligible patients in the case and control groups, respectively. The mean±SD 6-hour urine output in the first 5 days after surgery was 2586±725 mL in the control group and 2764±758 mL in the case group (p=0.31). The mean±SD serum creatinine level at the end of the 5th post-operative day was 1.3±0.5 and 1.4±0.7 mg/dL in the case and control groups, respectively (p=0.56). Serum creatinine level did not reduce to 1.5 mg/dL or lower in 6 of 36 control subjects and in 4 of 34 cases at the end of the 5th day (p=0.558). The mean±SD time to creatinine level <1.5 mg/dL was 1.3±1 days in the control group and 1.7±0.8 days in the case group (p=0.635). Hyperkalemia occurred in 3 of 36 patients in the control group and in 2 of 34 patients in the case group (p=0.318). The incidence of hyponatremia in the control group was 11% (4 of 36 patients) *vs* no patients in the case group (p=0.115).

Conclusion: Either half-saline or normal saline and 1/3–2/3 intravenous solution can be safely used as fluid replacement therapy after kidney transplantation.

## INTRODUCTION

Renal transplantation is the preferred treatment for patients who suffer from end-stage renal disease (ESRD). As much transplantation occurs, multitude number of complications such as hemodynamic instability, acid-base imbalances and electrolyte disturbances due to impaired renal function encounters [1]. Optimal graft perfusion and function after renal transplantation require sufficient intravascular volume repletion but there is no universally recommended regimen for fluid administration in patients undergoing kidney transplantation. Isotonic crystalloid solutions are the most commonly used fluids for volume restoration after renal transplantation, but they can impair electrolyte and acid-base balance [1, 2]. Normal saline has a much lower pH, but its higher sodium concentration and osmolality compared to plasma can lead to hyperchloremic metabolic acidosis and potential hyperkalemia, if administered in large amounts [3-8]. Half-saline solution has a lower sodium and chloride concentration and lower osmolality compared to normal saline. Although hyperchloremic metabolic acidosis is less common during administration of half-saline, theoretically, dilutional hyponatremia may occur [3]. However, considering that urinary concentration of sodium of newly transplanted diuresing kidney is typically 60 to 80 mEq/L, half-saline solution would restore the lost volume and electrolytes appropriately after transplantation [9].

To the best of our knowledge, no study has been performed to compare different post-operative intravenous fluid therapies for renal transplant recipients. Therefore, we conducted this trial comparing urine output, renal function, metabolic acidosis and electrolyte disturbances in renal transplant recipients treated with normal saline plus 1/3–2/3 *vs*. half-saline solutions.

## PATIENTS AND METHODS

All patients who had undergone kidney transplantation between June 21, 2008 and March 20, 2010 in Ahwaz Golestan Hospital were enrolled into this single-blind randomized clinical trial. Regarding to intravenous fluid replacement therapy, we randomly allocated patients into two groups: Group A patients (case) received half-saline solution, and group B patients (control) received normal saline plus 1/3–2/3 solutions. According to our center protocol, we replaced as much as 100% of hourly urine output in the first post-operative day, followed by 90% and 70% of every 2-hour urine output in the 2^nd^ and 3^rd^ days, respectively. In the 4^th^ and 5^th^ days, infusion of fluids was tapered to 50% and 30% of every 6-hour urine output, respectively. We continued immunosuppressive therapy for both groups based on the transplant center instructions.

After grafting, blood pressures and pulse rates were recorded each hour for two days and then every two hours. Plasma sodium, potassium and pH were measured every four hours. Blood samples were analyzed for plasma creatinine level, at least every 12 hours. Based on the physician’s judgment, patients were treated for hyperkalemia and/or metabolic disturbances. We excluded patients with acute or hyperacute rejection. Patients ended the trial when severe hyponatremia or symptomatic hyponatremia, hyperkalemia, metabolic acidosis, and/or need for dialysis occurred. We defined severe hyponatremia as a serum Na concentration <120 mEq/L, hyperkalemia as serum K level >6 mEq/L, and metabolic acidosis as pH<7.2 and/or serum bicarbonate level <15 mEq/L.

The primary outcome in this study was the mean 6-hour urine output. Others included 5^th ^day creatinine concentration, metabolic acidosis, potassium concentration and the need for dialysis in the first five days after grafting.

Data are presented as mean±SD or percentage as appropriate. χ^2^ or Fisher’s exact tests were used for comparisons of categorized variables. Comparison of the mean between the two groups was performed using independent-sample *Student’s t* test. A p <0.05 was considered statistically significant.

## RESULTS

Seventy-five patients were enrolled and randomized to receive either normal saline and 1/3–2/3 solutions or half-saline solution. Severe hyponatremia occurred in one patient in the control group. Four patients needed dialysis (3 in the control and one in the case groups, p=0.297); so they were excluded from the study. Finally, 36 and 34 eligible patients in the control and case groups completed the study. Demographic characteristics of either donors or recipients were comparable in both groups.

The mean±SD 6-hour urine output in the first five days after graft was 2586±725 mL in the control group and 2764±758 mL in the case group (p=0.31).

The mean±SD serum creatinine level at the end of 5^th ^day of operation was 1.4±0.73 and 1.3±0.51 mg/dL in the control and case groups, respectively (p=0.56). The plasma creatinine level did not reduce to 1.5 mg/dL or lower in 6 (17%) of 36 control subjects, and in 4 (12%) of 34 cases at the end of 5^th^ post-operative day (p=0.558). The mean±SD time to attain plasma creatinine level <1.5 mg/dL was 1.3±1 days in the control group and 1.7±0.85 days in the case group (p=0.635). Hyperkalemia occurred in 3 (8%) of 36 patients in the control group and in 2 (6%) of 34 patients in the case group (p=0.318). Four (11%) of 36 patients in the control group and no one in the case group developed hyponatremia (p=0.115).

## DISCUSSION

In spite of the fact that composition of intravenous fluid may have an impact on renal function in kidney transplant recipients, so far few studies have been performed to compare the effect of various solutions [3, 12]. We did not find any previous study comparing post-operative infusion of normal saline plus 1/3–2/3 solutions *vs*. Half-saline in renal transplant recipients. Some of preceding trials conducted by anesthesiologists evaluated only the differences between various fluids during the surgery and all of the patients received dextrose 5% and half-saline post-operatively. Thus, judgment about the distinction of intravenous fluid use after renal transplantation was impossible according to these surveys [1, 10, 11]. In our study, the mean 6-hour urine output, mean plasma creatinine level on the 5^th^ post-operative day, and mean time to attain a serum creatinine <1.5 mg/dL was similar in both groups. None of the patients experienced metabolic acidosis. The incidence of hyperkalemia was also not different in terms of the type of intravenous fluid administered. Neither normal saline, nor half-saline had any unfavorable consequences on renal function. Similar to our study, O’Malley, *et al.*, randomly allocated renal transplant recipients to receive normal saline or lactated Ringer’s solution and found that the type of administered fluid, did not affect the concentration of creatinine on the 3^rd^ post-operative day. Normal saline had no adverse effects on renal function. However, lactated Ringer’s solution was less associated with hyperkalemia and metabolic acidosis compared to normal saline [10]. The difference between the two studies in terms of the incidence of hyperkalemia and metabolic acidosis is most likely due to infusion of large amounts of normal saline during the short period of surgery in the O’Malley study. Development of hyperkalemia in normal saline-treated patients is presumably mediated through an extracellular shift of potassium caused by acute changes in plasma hydrogen ion concentration, which occurs in association with hyperchloremic metabolic acidosis.

Our study had some limitations. We did not measure plasma chloride, so definite diagnosis of hyperchloremic metabolic acidosis was not possible.

We concluded that both regimens, either normal saline combined with 1/3–2/3 solutions or half-saline alone can be used safely as post-operative intravenous fluid replacement in renal transplant recipients. However, it seems that use of half-saline solution is easier, particularly in diabetic patients for lack of glucose.
